# Endometrial Cancer in Aspect of Forkhead Box Protein Contribution

**DOI:** 10.3390/ijerph191610403

**Published:** 2022-08-21

**Authors:** Olga Adamczyk-Gruszka, Agata Horecka-Lewitowicz, Jakub Gruszka, Monika Wawszczak-Kasza, Agnieszka Strzelecka, Piotr Lewitowicz

**Affiliations:** 1 Department of Gynaecology and Obstetrics, Collegium Medicum, Jan Kochanowski University, 25-369 Kielce, Poland; 2 Department of Obstetrics and Gynaecology, Province Hospital, 25-369 Kielce, Poland; 3 Institute of Health Sciences, Jan Kochanowski University, 25-369 Kielce, Poland; 4 2nd Department of Obstetrics and Gynaecology, Medical University of Warsaw, 02-091 Warsaw, Poland; 5 Department of Surgical Medicine with the Laboratory of Medical Genetics, Institute of Medical Sciences, Jan Kochanowski University, 25-369 Kielce, Poland; 6 Department of Clinical and Experimental Pathology, Institute of Medical Sciences, Jan Kochanowski University, 25-369 Kielce, Poland

**Keywords:** endometrial cancer, microsatellite instability, immunohistochemistry, Illumina sequencing

## Abstract

(1) Background: The present study aimed to investigate the influence of forkhead box (FOX) on endometrial cancer (EC) progression. For a better understanding, the driving mechanisms are vital to identifying correlations between genes and their regulators. (2) Methods: The study enrolled one hundred and three white female patients with confirmed EC. For the analysis, we used next-generation sequencing with the Hot Spot Cancer Panel provided by Illumina Inc., San Diego, CA, USA, and an immunohistochemical analysis of FOXA1, FOXP1, and estrogen receptors. (3) Results: FOXA1 silencing led to a worse outcome based on the correlation with FOXA1 (test log-rank *p* = 0.04220 and HR 2.66, *p* = 0.033). Moreover, FOX proteins were closely correlated with TP53 and KRAS mutation. (4) Conclusions: Our study confirmed previous reports about FOX box protein in the regulation of tumor growth. A remarkable observation about the unclear crosstalk with crucial genes, as TP53 and KRAS need deeper investigation.

## 1. Introduction

In past decades, morbidity and mortality associated with endometrial cancer (EC) increased despite diagnostic progress. Obesity is particularly associated with EC, and EC is prevalent in high-income countries owing to their associated high rate of obesity [1]. Currently, the Cancer Genome Atlas (TCGA) proposed a molecular subgrouping of EC that mechanically separates high- and low-grade tumors [2]. Afterward, updated diagnostical algorithms were incorporated. Looking from the daily practice point of view, this molecular subgrouping would be cumbersome for patients’ management but a wide availability of molecular tests and lowering prices inspires hope for a change. Essentially, for integrated ‘histomolecular’ diagnosis, pathology reports should include information about the mutational status of the POLE gene and perform at least the immunohistochemical tests of p53, MSH6, and PMS2 [3].

FOXA1 and FOXP1 are members of the forkhead box (FOX) family of transcription factors, formerly known as the hepatocyte nuclear factor (HNF) family. Recent studies have shown that the FOX transcription factor family is closely associated with hormone-dependent carcinogenesis, especially with steroid receptors [4,5,6].

The role of FOXA1 in cancer remains not fully defined, as both pro- and anti-tumorigenic functions have been uncovered. Research has identified that FOXA1 and FOXA2 expression inversely correlates with disease progression and/or aggressiveness; for example, FOXA1 and FOXA2 are readily detected in normal epithelium and precancerous lesions, but the expression is commonly lost in poorly differentiated disease (and event associated with EMT-like phenotypes) [6]. In addition, FOXA1 has been identified as a key transcription factor that binds to the promoters of more than 100 genes, in turn regulating many cellular functions. FOXA1 proteins bind to DNA and induce nucleosomal rearrangement, often resulting in an open chromatin structure [7,8]. The process facilitates the additional binding of additional transcription factors, which include the estrogen receptor subunit α [9]. FOXA1 expression is significantly correlated with androgen receptors (AR) expression in the hormone-dependent tissues via the Notch route activation. Moreover, it was postulated that high levels of FOXP1 positively correlated with the survival rate of infiltrative breast cancer [10,11,12]. The Notch pathway is initiated by ligand binding, followed by intramural proteolytic cleavage of the Notch1 receptor to release the active form of the Notch intracellular domain (NICD). The NICD transfers into the nucleus and acts as a transcription activator to increase the target genes’ expression, such as the Hairy enhancer of split1 [13]. However, the main fields of interest of the FOX protein are prostate and breast cancer, and the abnormal activation of the Notch pathway, which promotes proliferation in EC [12,14]. The association between hormone receptors and prognostic variables (FIGO stages, histological degree, and survival) has been well documented for EC [10,11,12]. FOXA1 levels are associated with good or poor prognosis depending on the patient group and have been proposed as a marker associated with survival in hormone-dependent cancers [15,16]. For over a decade, crosstalk between the Notch pathway and other crucial controlling genes has been reported. The overactivity of Notch reduces the p53 quantity through increased activity of MDM2, an E3 ubiquitin ligase that targets p53 for degradation. The underlying mechanism involves decreased expression of ARF (encoded by *CDKN2A*), a key negative regulator of MDM2 activity. Importantly, tumorigenesis in this system depends on sustained Notch activity, as attenuation of the Notch expression dramatically increases p53 levels and tumor regression through apoptosis [17]. This inspired us to study EC in this aspect.

The purpose of the present study was to explore the expression of FOX protein, and its influence on EC molecular basis and cancer progression.

## 2. Materials and Methods

A total of one hundred and three white female patients with confirmed EC were enrolled. All patients underwent surgery and other oncological procedures between 2005 and 2017. The collective evaluation data and follow-up data were tabulated. To align tumor staging, each case was re-diagnosed according to the Eighth Edition of TNM Classification [18]. Detailed characteristics of the group have been described in our previous publication [19].

All participants underwent surgical treatment without previous radio-chemotherapy to conduct a credible comparative analysis of tumor characteristics, the treatment, and unchanged molecular profiling.

To achieve this goal, we used the Hot Spot Cancer Panel provided by Illumina Inc., San Diego, CA, USA. The gene battery comprised BL1, EGFR, GNAS, KRAS, PTPN11, AKT1, ERBB2, GNAQ, MET, RB1, ALK, ERBB4, HNF1A, MLH1, RET, APC, EZH2, HRAS, MPL, SMAD4, ATM, FBXW7, IDH1, NOTCH1, SMARCB1, BRAF, FGFR1, JAK2, NPM1, SMO, CDH1, FGFR2, JAK3, NRAS, SRC, CDKN2A, FGFR3, IDH2, PDGFRA, STK11, CSF1R, FLT3, KDR, PIK3CA, TP53, CTNNB1, GNA11, KIT, PTEN, and VHL. The sequencing target was 2800 COSMIC mutations from 50 oncogenes and tumor suppressor genes.

In addition, we performed tissue microarray (TMA Master II, 3Dhistech, Budapest, Hungary) for immunohistochemistry. We performed the immunohistochemical assays using the automated IHC/ISH slide staining system BenchMark Ultra (Ventana Medical Systems; Roche Group, Tucson, AZ, USA). After deparaffinization and rehydration of the samples, we performed the unmasking processes using CC1 (Ventana Medical Systems; Roche Group, Tucson, AZ, USA) and incubation with primary antibodies (time and temperature of both antigen retrieval and primary antibody incubation followed the manufacturer’s recommendations). In addition, further routine steps were performed. Moreover, we used the Ventana ultra-View Universal DAB Detection Kit and Opti View Detection Kit. The immunohistochemistry details are presented in Table 1.

### 2.1. Molecular Analysis

DNA isolation: cancer genomic DNA was extracted from formalin-fixed paraffin-embedded tissue using the MagCore^®^ Genomic DNA FFPE One-Step Kit (RBC Bioscience, Taiwan, China). The quality was quantified using DeNovix DS-11 Spectrophotometer (DeNovix, Wilmington, DE, USA) and QuantiFluo^®^ ONE dsDNA System (Promega, WI, USA).

The assay generated a library of 207 gene-specific amplicons and targeted ~2800 clinically relevant mutations.

Sequencing: the products were analyzed by next-generation sequencing (NGS) using the Illumina platform, MiSeq Dx.

Data analysis: an analysis of the NGS data was performed using the GALAXY platform (usegalaxy.org). Sequencing reads (FASTQ files) were aligned to the human reference genome hg19 using the Bowtie2 tool. Variant calling was performed using the Varscan2 tool. Parameters used for the analysis were minimum allele frequency—0.05, minimum quality—20, and minimum coverage ×80. All variants were annotated with ANNOVAR (Available online: https://wannovar.wglab.org, accessed on 2 May 2022). The results were visualized using the R Bioconductor package Maftools (Available online: http://bioconductor.org/, accessed on 2 May 2022) [19].

### 2.2. Ethical Statement

This study used human tissues for the experiment, performed in concordance with the ethical standards of the Declaration of Helsinki of the updated version, 2004. In addition, the study was approved by the Ethical Commission of the Faculty of Medicine and Health Science, the Jan Kochanowski University in Kielce, Poland, in 2019.

### 2.3. Statistical Analysis

Descriptive statistics were performed to summarize the data in a manageable form. Quantitative data were reported as mean, standard deviation, median, and range. Categorical data were expressed as number and percentage distributions. The Chi-square test or Fisher’s exact test was applied to compare proportions, and a multivariable logistic regression model was used to assess the relationship between the targeted genes. The follow-up period was calculated as the number of years from the date of surgery to disease recurrence. The death that occurred from causes other than cancer was recorded. The last contact with the patient was also presented. The univariate associations between disease-free survival in selected patients and tumor characteristics were evaluated using the univariate Cox proportional-hazards model. Analyses of continuous variables were dichotomized in the median. To identify the independent prognostic factor for disease-free survival, a Multivariate Cox proportional-hazards model with backward selection (with a cut-off of 0.15) was performed on variables that were statistically significant in univariate analysis.

All statistical tests were two-sided, and values <0.05 were considered significant. Computations were performed using STATISTICA (data analysis software system), StatSoft, Inc. (2014), version 12, Tulsa, OK, USA, Available online: http://www.statsoft.com/, accessed on 2 May 2022.

## 3. Results

The estimation of targeted protein frequency was conducted (Table 2), revealing that FOXA1 occurred in 24 cases, especially in the FIGO IA stage.

**Figure 1:**
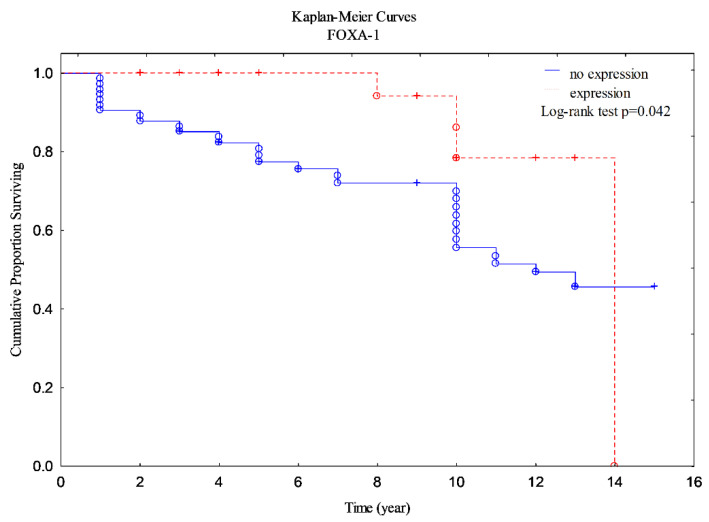
The Kaplan–Meier curves for FOXA1 (*p* = 0.042).

A detailed OS analysis was conducted to investigate the impact of the clinical outcome. The Cox univariate analysis provided us with significant results concerningFOXA1 (test log-rank for FOXA1 2,031559, *p* = 0.04220 (Figure 1) and HR 2.66, *p* = 0.033), revealing that FOXA1 silencing led to a poor outcome. The correlation was found between FOXA1 and FOXP1 (R = 0.2872 *p* = 0.0041). We noted the correlation of OS with FOXP1 (*p* > 0.05).

The next stage of our research was a confrontation of FOX protein with tested genes. A general mutational contribution was as follows: PTEN 49%, PIK3CA 35%, KRAS 25%, TP53 20%, FGFR-2 14%, CTNNB1 12%, FBXW7 9%, ATM 1%, ALK1 1%, and APC 1%.

Interestingly, we noted the coincidence of a lack of FOXP1 with TP53 mutation (R = 0.31, *p* = 0.0106) and FOXA1 with KRAS mutation (R = −0.246, *p* = 0.0446). This information confirmed the impact on advanced-stage FIGO (FOXP1 R = 0.2379 *p* = 0.018924, FOXA1 R = −0.2643 *p* = 0.0088). The multivariate Cox models covering all tested genes and FOX proteins unveiled prognostic values for TP53 (HR = 0.12, *p* = 0.011), KRAS (HR = 0.31, *p* = 0.023), and lack of FOXA1(HR = 0.19, *p* = 0.017).

Estrogen receptor expression was detected in all FOXP1-positive cases and more than 90% of FOXA1-positive ones.

## 4. Discussion

A remarkable change in the molecular subgrouping of EC has been witnessed in the present years. Presently, extracting low- and high-grade EC has been proposed, where the latter was driven by TP53 mutation, high copy number variations, and microsatellite instability. This study attempts to discuss an internal crossing pathway involved in EC progress. A recent report by Cruz et al. depicts a connection between FOX protein and beta-catenin in an experimental model of breast cancer. Moreover, the potential clinical significance of JAM-A-dependent regulation of FOXA1 is important because of the FOXA1 connection with endocrine resistance. FOXA1 expression has been shown to be positively associated with estrogen receptor (ER)+ breast cancer. In our cohort study, we recorded over 90% concordance in co-expression with ER and FOXA1. Our results confirm a protective effect of ER/FOXA1 co-expression [20,21,22]. Our linear and combine statistics proved the prognostic value of FOXA1. Our results confirmed previous data and added new information regarding the correlation with crucial genes worsening prognosis. The Notch pathway is believed to be an emerging target for cancer therapy. It is vividly observed in neuroendocrine and lymphoid malignancies with delta-like ligand 3(DLL3) [23].

The clinical impact of forkhead protein box FOXP1 is still unclear. We did not observe the significance of FOXP1; however, another report suggested that high nuclear expression of FOXP1 would be a recent event in EC carcinogenesis. Like the FOXA1, a tight connection with ER and AR was also noted [24]. The abovementioned crosstalk between pathways, especially Notch, is in the spotlight of modern oncology. A recent study focused on pancreatic cancer in the crossing signaling routes, which included prometastatic, pioneer transcription factor FOXA1. An activation of transcriptional ways starts pro-proliferative cascades of the Wnt pathway or MAPK [25].

Our study noted a 25% frequency of KRAS mutation and 20% TP53 mutation. Moreover, a lack of FOXP1 was correlated with TP53 mutation (*p* = 0.0106), revealing a correlation of FOXA1 with KRAS mutation (*p* = 0.0446).

The animal experimental models provide only random information, and there is a lack of information on EC contribution, leading to the investigation of other organ-specific tumors. For example, the loss of FOXA1 increased the level of transforming growth factor-beta 3 (TGFβ3) and activated TGFβ signaling pathway in prostate cancer [26]. More interestingly, the deletion of FOXA1 in KRAS-driven neoplasia derived from alveolar cells resulted in keratinizing squamous cell carcinoma, indicating that FOXA1/2 manipulated the growth of lung cancer in a context-dependent manner [27]. With the crucial roles of FOXA in tumorigenesis and hormone-dependent cancers, numerous efforts have been devoted to targeting FOXA for cancer therapies. Unfortunately, no specific strategy has been recorded. In tamoxifen-resistant ER+ breast cancer, the high level of FOXA1 could be reduced by sorafenib and nilotinib, leading to the re-sensitization of the tamoxifen-resistant cells [28].

Because Notch signaling is observed in many malignancies, there is hope for universal treatment for improving clinical survival.

## 5. Conclusions

Our study confirmed previous reports about the FOX box protein in the regulation of tumor growth. A remarkable observation about the unclear crosstalk with crucial genes, as TP53 and KRAS need additional research.

This study was limited by the population selected from one region in Poland, and the focused follow-up time varied among the same participants. In future research, we plan to add a control group. Moreover, our gene panel did not cover the POLE gene. The strengths of this work include the long-term observation and the use of modern methods of genetic testing.

## Figures and Tables

**Table 1:** The antibodies characteristics.

	Clone	Dilution	Manufacturer	Positive Expression
ER	SP1 monoclonal, rabbit	1 µg/mL; ready to use	Ventana	nuclear
FOXA1	2F83 monoclonal; mouse	0.72 µg/mL; ready to use	Cell Marque	nuclear
FOXP1	SP133 monoclonal; rabbit	0.8 µg/mL; ready to use	Cell Marque	nuclear

**Table 2:** Expression of targeting proteins according to grade and stage.

N = 103	Number of Positive Cases	G			FIGO					FOXA1	FOXP1	ER
		G1	G2	G3	IA	IB	II	III	IV			
FOXA1 (24%)		10	13	1	22	2	0	0	0		0	21
FOXP1(8%)		2	5	1	3	1	4	0	0	0		7
ER (77%)		42	33	5	54	10	12	2	1	21	7	

## Data Availability

Data will be available from the corresponding author upon reasonable request.
